# *In vitro* antibacterial activity of antiretroviral drugs on key commensal bacteria from the human microbiota

**DOI:** 10.3389/fcimb.2023.1306430

**Published:** 2024-01-08

**Authors:** Elisa Rubio-Garcia, Núria Ferrando, Núria Martin, Clara Ballesté-Delpierre, Jose M. Miró, Roger Paredes, Climent Casals-Pascual, Jordi Vila

**Affiliations:** ^1^ Department of Clinical Microbiology, Centre de Diagnòstic Biomèdic (CDB), Hospital Clínic of Barcelona, Barcelona, Spain; ^2^ Molecular Core Facility, Centre de Diagnòstic Biomèdic (CDB), Hospital Clínic of Barcelona, Barcelona, Spain; ^3^ Departament de Fonaments Clínics, Facultat de Medicina i Ciències de la Salut, Universitat de Barcelona, Barcelona, Spain; ^4^ Barcelona Institute for Global Health (ISGlobal), Barcelona, Spain; ^5^ Infectious Disease Networking Biomedical Research Center, Centro de Investigación Biomédica en Red de Enfermedades Infecciosas (CIBERINFEC), Instituto de Salud Carlos III, Madrid, Spain; ^6^ Infectious diseases Service. Hospital Clínic-IDIBAPS. University of Barcelona, Barcelona, Spain; ^7^ Fundació Lluita Contra les Infeccions, Department of Infectious Diseases, Hospital Germans Trias i Pujol, Badalona, Barcelona, Spain; ^8^ IrsiCaixa AIDS Research Institute, Badalona, Catalonia, Spain; ^9^ Center for Global Health and Diseases, Department of Pathology, Case Western Reserve University School of Medicine, Cleveland, OH, United States

**Keywords:** antiretroviral therapy, antibacterial activity, human commensal bacteria, multidrug resistant bacteria, elvitegravir repurposing

## Abstract

**Introduction:**

Antiretroviral therapy has improved life expectancy in HIV-infected patients. However, people living with HIV under antiretroviral therapy are at higher risks of developing chronic complications and acquiring multidrug resistant bacteria than healthy population. These factors have been associated with shifts in gut microbiome composition and immune activation. It is unclear how antiretroviral drugs affect gut microbiota composition, but it has been observed that antiretroviral treatment is not able to fully restore gut health after HIV infection. Additionally, some antiretroviral drugs have shown antibacterial activity suggesting that these drugs could have a direct impact on the human microbiome composition.

**Methods:**

We determined the *in vitro* antibacterial activity of 16 antiretroviral drugs against a set of key clinically relevant and human commensal bacterial strains.

**Results:**

Our results demonstrate that 5 antiretroviral drugs have *in vitro* antibacterial activity against gut and vaginal human commensal bacteria. Zidovudine has antibacterial activity against *Escherichia coli*, *Klebsiella pneumoniae* and *Prevotella bivia*, abacavir against *Gardnerella vaginalis*, efavirenz against *G. vaginalis* and *P. bivia* and bictegravir against *Enterococcus* spp. and *G. vaginalis*. Moreover, we describe for the first time that elvitegravir has antibacterial activity against *G. vaginalis* and *P. bivia* and, most importantly, against vancomycin-resistant *Enterococcus* spp. and methicillin-resistant *Staphylococcus aureus* strains with MIC values of 4-16 and 4 µg/mL, respectively showing high level of effectiveness against the tested multidrug-resistant bacteria.

**Discussion:**

Our results underscore that some antiretroviral drugs may influence the human microbiota composition. In addition, we report the potential use of elvitegravir to treat multidrug-resistant Gram-positive bacteria warranting the need of clinical studies to repurpose this antiretroviral drug.

## 1 Introduction

There are more than 37 million people living with HIV-1 (PLWH) in the world, but all human beings are at potential risk of acquiring HIV-1 at some point in their lifetime. Despite general progress in antiretroviral (ARV) treatment-based strategies to prevent and treat HIV-1 infection, every year nearly 2 million new HIV-1 infections and more than 0.5 million deaths due to AIDS-related causes continue to occur (World Health Organization, 2020; Unaids, 2022).

Antiretroviral treatment (ART) is effective in virtually all patients chronically infected with HIV-1, improving life expectancy and quality of life. However, some PLWH under ARV treatment end up developing chronic clinical complications, which have been related to chronic inflammation, immune activation and changes in the intestinal microbiome composition (Bandera et al., 2017; Guillén et al., 2019). Several studies have observed differences in the composition of the gut microbiome between PLWH on ART and untreated subjects with HIV. However, the gut microbiome of PLWH on ART remains different from that of healthy patients, indicating that ARV treatment is not able to fully restore gut health after HIV infection (Li et al., 2016; Pinto-Cardoso et al., 2018).

Additionally, PLWH are at higher risk of acquiring and being infected by multidrug-resistant (MDR) microorganisms. This is because they are more likely to present known risk factors for MDR microorganism acquisition, including more hospital admissions, higher rates of antibiotic intake, the previously mentioned chronic clinical complications and changes in the intestinal microbiome composition (Olaru et al., 2021a; Olaru et al., 2021b; Henderson et al., 2022). The emergence and dissemination of MDR microorganisms is an important public health problem due to high morbidity and mortality of infections caused by these agents (Huemer et al., 2020). Moreover, the development of new antibiotics has dropped considerably in recent years leaving limited effective treatment options against MDR bacteria (Aira et al., 2019).

Some antiretroviral drugs have shown to have antibacterial activity (Shilaih et al., 2018; Ray et al., 2021) and, therefore, these drugs could have a direct impact on the disruption of the intestinal microbiota and the selection of MDR microorganisms. Considering that over 28 million people worldwide receive ART, which is a lifelong treatment, there is a constant ART selective pressure on gut microbiota (Unaids, 2022). This continuous selective pressure could have significant implications on the emergence and dissemination of MDR organisms, ultimately leading to clinical consequences related to resistance induction. Consequently, it is worth to thoroughly characterize these effects, but the antibacterial activity of antiretroviral drugs has not been analysed against a sufficient variety of strains representative of the human intestinal microbiota.

In this work, we therefore aimed to evaluate the *in vitro* antibacterial effect using the broth microdilution method of 16 antiretroviral drugs representative of 4 different antiviral drug families and the booster cobicistat in a set of 8 different reference bacterial strains and 126 clinical isolates representative of the human commensal microbiota and common human pathogens. The set of selected bacterial strains included Gram-positive and Gram-negative bacteria as well as clinical strains showing different antibiotic resistance profiles.

## 2 Materials and methods

### 2.1 Antiretroviral drugs and antibiotics

A set of antiretroviral drugs representative of each antiretroviral class were included (**Table 1**) in the study. All antiretroviral drugs were purchased from Merck Life Science (Madrid, Spain), except for Dolutegravir (DTG), Doravirine (DOR), Rilpivirine (RIL) and Cobicistat (COB) which were purchased from Quimigen (Madrid, Spain) and Tenofovir alafenamide (TAF), Bictegravir (BIC) and Elvitegravir (EVG) which were kindly provided by Gilead Sciences Inc. (Foster City, California, USA). Stock solutions of each ARV were prepared using dimethyl sulfoxide (DMSO), deionized water or methanol according to each ARV solubility (**Table 1**). Additionally, active antibiotics against the analyzed bacterial strains including Colistin, Gentamicin and Clindamycin were purchased from Quimigen (Madrid, Spain).

**Table 1:** Minimum Inhibitory concentration (MIC) of 17 antiretrovirals against a set of reference bacterial strains.

	MIC (µg/mL)								
	Solvent	*Escherichia* *coli*	*Klebsiella* *pneumoniae*	*Enterococcus* *faecalis*	*Acinetobacter* *baumannii*	*Pseudomonas aeruginosa*	*Gardenerella vaginalis*	*Bacteroides thetaiotaomicron*	*Prevotella* *bivia*
Nuceloside Reverse-Trasncriptase Inhibitors									
Tenofovir disiproxil fumarate (TDF)	H_2_O	>128	>128	>128	>128	>128	>128	>128	>128
Tenofovir alafenamide (TAF)	DMSO	>128	>128	>128	>128	>128	>128	>128	>128
Abacavir Sulfate (ABC)	H_2_O	>128	>128	>128	>128	>128	**32-128**	>128	>128
Lamivudine (3TC)	H_2_O	>128	>128	>128	>128	>128	>128	>128	>128
Zidovudine (AZT)	H_2_O	**1-2**	**0.5-1**	>128	>128	>128	>128	>128	**8**
Emtricitabine (FTC)	H_2_O	>128	>128	>128	>128	>128	>128	>128	>128
Non-Nuceloside Reverse-Transcriptase Inhibitors									
Efavirenz (EFV)	Methanol	>128	>128	>128	>128	>128	**64-128**	>128	**64**
Nevirapina (NVP)	DMSO	>128	>128	>128	>128	>128	>128	>128	>128
Rilpivirine (RIL)	H_2_O	>128	>128	>128	>128	>128	>128	>128	>128
Doravirine (DOR)	DMSO	>128	>128	>128	>128	>128	>128	>32^a^	>128
Protease Inhibitors									
Darunavir (DRV)	DMSO	>128	>128	>128	>128	>128	>128	>128	>128
Atazanavir Sulfate (ATV)	DMSO	>128	>128	>128	>128	>128	>128	>128	>128
Integrase strand transfer Inhibitors									
Raltegravir potassium (RAL)	H_2_O	>128	>128	>128	>128	>128	>128	>128	>128
Bictegravir (BIC)	DMSO	**128**	**128**	**64**	>128	>128	**128**	>128	>128
Elvitegravir (EVG)	DMSO	>128	>128	**8-16**	>128	>128	**16-32**	>128	**32**
Dolutegravir (DTG)	DMSO	>128	>128	>128	>128	>128	>128	>128	>128
Booster									
Cobicistat (COB)	DMSO	>128	>128	>128	>128	>128	>128	>128	>128
Antibotics used as positive controls									
Colistin	H_2_O	0.25-1	0.5-1		2-4	1-2			32
Gentamicin	H_2_O			8-32			32-64		
Clindamycin	H_2_O							4-8	
Solvents^b^									
DMSO		12.5%	12.5%	12.5%	6.25%	12.5%	>25%	25%	25%
Methanol		25%	25%	12.5%	12.5%	25%	12.5-25%	12,5%	6.25%

^a^Higher ARV concentrations could not be tested due to technical problems. ^b^The proportion of each solvent for diluting the drugs are presented as volume percent (v/v).

Bold indicates compounds with observed MIC at the given concentration. Solvent used for each antiretroviral and antibiotic used as positive control for each strain are indicated. Reported values are the range of four replicates (two biological replicates and two technical replicates).

### 2.2 Bacterial strains

Bacterial strains issued from the American Type Culture Collection (ATCC) were used for a first antibacterial activity screening of the tested antiretroviral (ARV) drugs. For this screening, antibiotic susceptible strains were used, namely *Escherichia coli* ATCC 25922, *Klebsiella pneumoniae* ATCC 35657, *Acinetobacter baumannii* ATCC 17978, *Pseudomonas aeruginosa* ATCC 9027, *Enterococcus faecalis* ATCC 35657, *Gardnerella vaginalis* ATCC 14018 and *Bacteroides thetaiotaomicron* ATCC 29741. Additionally, a clinical isolate of *Prevotella bivia* was also tested.

The antiretroviral drugs identified as having antibacterial activity in the first screening were further validated using a set of 126 clinical strains showing different antibiotic resistance profiles. The selected bacterial strains were 20 *Escherichia coli* [30% (6/20) producing extended-spectrum β-lactamase (ESBL) and 25% (5/20) producing a carbapenemase], 17 *Klebsiella pnuemoniae* [29% (5/17) producing ESBL and 41% (7/17) producing carbapenemase], 22 *E. faecium, 16 E. faecalis*, 22 *Staphylococcus aureus* [45.45% (10/22) methicilin-resistant *S. aureus* (MRSA)], 16 *G.* vaginalis and 13 P*. bivia*. The antibiotic resistance profiles and sample origin of the selected clinical bacterial strains are shown on **Supplementary Tables (S1–S7)**. Antibiotic resistance profiles for *G. vaginalis* and *P. bivia* were not available as they were not routinely tested.

### 2.3 *in vitro* susceptibility testing: MIC determination

The minimal inhibitory concentrations (MICs) of each antiretroviral drug against the selected bacterial strains were determined by the broth microdilution method following Clinical and Laboratory Standards Institute (CLSI) guidelines (Carpenter et al., 2018; Weinstein et al., 2018). CLSI guidelines for anaerobic bacteria were followed for *B. thetaiotaomicron*, *P. bivia* and *G. vaginalis* strains. Briefly, bacterial strains were inoculated in Blood Agar or Brucella agar for anaerobic bacteria and incubated at 37°C for 16-20h or for 44-48h under anaerobic atmosphere for anaerobic bacteria. Grown bacteria were resuspended in saline (0.9% sodium chloride) to obtain 0.5 McFarland turbidity standard, followed by a 100-fold dilution in Cation adjusted Mueller Hinton Broth (CAMHB) for aerobic bacteria and Brucella broth supplemented with hemin (5 μg/mL), vitamin K1 (1 μg/mL), and lysed horse blood (5%) for anaerobic bacteria.

Stock solutions of each antiretroviral drug were diluted in deionized water to a concentration of 1024 µg/mL and further dilutions were performed using CAMHB or supplemented Brucella broth. The solvents methanol and DMSO were tested separately, and they did not affect bacterial growth at the assay conditions (≤5.12% v/v) (**Table 1**). Together, a final bacterial suspension of 7.5x10^5^ CFU/mL was inoculated in a 96-well plate and the tested two-fold serial dilution range concentration for each ARV drug was 128-0.125 μg/mL. Wells containing no ARV and wells containing no bacteria were included as growth and negative controls, respectively. Additionally, an active antibiotic against the bacterial strain was tested simultaneously as a positive control.

After incubation at 37°C for 18-24h for aerobic bacteria and 44-48h under anaerobic conditions for anaerobic bacteria MIC was determined visually as the lowest drug concentration that inhibited bacterial growth.

For the first antibacterial activity screening, all MICs were determined four times for each antiretroviral-bacteria combination (two biological and two technical replicates). For the validation of antiretroviral antibacterial activities using clinical strains, each MIC was determined once and MIC_50_ and MIC_90_, defined as the minimal inhibitory concentration of drug capable of inhibiting 50% and 90% of the clinical tested isolates, respectively, were calculated.

## 3 Results

### 3.1 Antibacterial susceptibility testing: Initial Screening

The initial screening for antibacterial activity of 16 antiretroviral drugs representative of each ART class and the booster cobicistat against a set of 8 bacterial strains representative of the human commensal microbiota and pathogenic strains is shown in **Table 1**. Five of the 17 compounds showed antibacterial activity within the tested concentration range. Among the Nucleoside Reverse-Transcriptase Inhibitors (NRTIs) class, Abacavir sulfate (ABC) showed activity against *G. vaginalis* with MIC values of 32-128 µg/mL and Zidovuidine (AZT) showed activity against *E. coli* and *K. pneumoniae* with MIC values of 1-2 and 0.5-1 µg/mL respectively. Among the Non-Nucleoside Reverse-Transcriptase Inhibitors class (NNRTIs), Efavirenz (EFV) showed activity against *G. vaginalis* with MIC values of 64-128 µg/mL. Regarding Integrase Inhibitors, Bictegravir (BIC) showed activity against *E. coli, K. pneumoniae, E. faecalis* and *G. vaginalis* with MIC values of 128, 128, 64 and 128 µg/mL respectively and Elvitegravir (EVG) showed activity against *E. faecalis* and *G. vaginalis* with MIC values of 8-16 and 16-32 µg/mL respectively. No antibacterial activity was identified for the booster cobicistat or other protease inhibitors such as darunavir and atazanavir sulfate.

### 3.2 Antibacterial activity validation with clinical strains

In order to confirm the antiretroviral antibacterial activity observed in the first screening, MIC was determined for a set of clinical strains. The evaluated antiretroviral-bacteria combinations were ABC against *G. vaginalis*, AZT against *P. bivia*, EFV against *G. vaginalis* and *P. bivia*, BIC against *E. coli, K. pneumoniae, E. faecalis* and *G. vaginalis*, and EVG against *E. faecalis*. Additionally, *E. faecium* clinical strains were included to test BIC and EVG antibacterial activity and *S. aureus* clinical strains to test EVG antibacterial activity in order to include clinically relevant Gram-positive bacteria. Zidovudine inhibitory effect against *E. coli* and *K. pneumoniae* was not further validated as this activity has been previously described in other studies (Elwell et al., 1987; Lewin et al., 1990). **Table 2** shows MIC_50_ and MIC_90_ values of the tested antiretrovirals against the selected clinical strains. Additionally, individual MIC values for each antiretroviral-clinical strain are shown in **Supplementary Tables**.

**Table 2:** Antibacterial activity of ABC, AZT, EFV and BIC antiretroviral against a set of clinical bacterial strains.

Clinical strains	Number of tested strains	*^a^* MIC_50_ (µg/mL)	*^b^* MIC_90_ (µg/mL)
Abacavir Sulfate (ABC)			
*Gardnerella vaginalis*	16	32	64
Zidovudine (AZT)			
*Prevotella bivia*	13	2	4
Efavirenz (EFV)			
*Gardnerella vaginalis*	16	32	32
*Prevotella bivia*	13	32	64
Bictegravir (BIC)			
*Escherichia coli*	20	>128	>128
*Klebsiella pneumoniae*	17	>128	>128
*Enterococcus faecium*	22	64	64
*Enterococcus faecalis*	16	64	64
*Gardnerella vaginalis*	16	128	128
Elvitegravir (EVG)			
*Gardnerella vaginalis*	16	16	16
*Prevotella bivia*	13	32	32
*Enterococcus faecium*	22	4	16
*Enterococcus faecalis*	16	4	16
*Staphylococcus aureus*	22	4	4

^a^MIC_50_ and ^b^MIC_90_ are defined as the minimal inhibitory concentration of drug capable of inhibiting 50% and 90% of the tested isolates, respectively.

The antibacterial activity of all antiretroviral-bacteria combinations was confirmed using clinical strains, except for BIC, as no activity against *E. coli* and *K. pneumoniae* clinical strains was observed at the tested concentration range (BIC showed activity against only 1/17 K*. pneumoniae* and 4/20 *E. coli* strains with 128 µg/mL MIC values). The antiretroviral showed lower MIC values against some clinical strains compared to the reference strains, but these differences were not higher than two-fold dilution in any case. Remarkably, BIC showed antibacterial activity against *E. faecium* clinical strains with MIC values of 64 µg/mL and EVG showed antibacterial activity against *E. faecium* and *S. aureus* clinical strains with MIC values of 4-16 and 4 µg/mL, respectively. Of note, these clinical strains included vancomycin-resistant enterococci (VRE) and methicillin-resistant *Staphylococcus* aureus (MRSA) strains. In all cases, the antiretroviral antibacterial activity was not associated to the clinical strain resistance mechanism or resistance profile to any of the tested antibiotics (**Supplementary Tables** **).**

## 4 Discussion

In this study we report the antibacterial activity of different antiretrovirals against a set of clinically relevant and human commensal bacterial strains. We demonstrate antibacterial activity of AZT against *E. coli, K. pneumoniae*, and *P. bivia*, ABC against *G. vaginalis*, EFV against *G. vaginalis* and *P. bivia* and BIC against *Enterococcus* spp. and *G. vaginalis*. Importantly, we report remarkable EVG antibacterial activity against *G. vaginalis* and *P. bivia* and most importantly against *Enterococcus* spp. and *Staphylococcus aureus* with MIC values of 4 µg/mL and including VRE and MRSA strains.

The antibacterial activity of AZT against different members of the *Enterobacterales* including *E. coli* and *K. pneumoniae* was already observed at the beginning of HIV pandemic (Elwell et al., 1987; Lewin et al., 1990). Zidovudine is a nucleoside analog which inhibits bacterial growth through DNA chain termination previous phosphorylation by thymidine kinases present in bacteria. Resistance to Zidovudine by mutations or loss of activity of bacterial thymidine kinases have been reported (Doléans-Jordheim et al., 2011) and Zidovudine-resistant bacterial strains have been isolated from fecal samples from AIDS patients receiving the drug (Lewin et al., 2006). Additionally, the use of AZT in combination with other antibiotics has been previously studied *in vitro* for the treatment of infections caused by MDR *Enterobacterales* (Ng et al., 2018; Falagas et al., 2019; Desarno et al., 2020; Antonello et al., 2021).

We also report AZT antibacterial activity against *P. bivia* with low MIC of 4 µg/mL, this finding agrees with Ray et al. (2021). who identified AZT activity against a set of clinical strains of *Prevotella* spp. obtaining similar MIC values. However, while Ray et al. (2021) also identified AZT activity against a set of clinical strains of *Bacteroides* spp., in our study AZT did not show activity against the reference strain *B. thetaiotaomicron*.

Zidovudine is currently not frequently prescribed in high-income countries as its use is limited as a second-line regimen or as prevention of mother-to-child transmission of HIV during delivery and first years of life. However, in low-income countries it is still widely used (World Health Organization, 2021; Palacios et al., 2023). Thus, AZT antibacterial activity could impact many PLWH worldwide. Additionally, the obtained AZT MICs are within the estimated AZT gut concentrations (Ray et al., 2021) at the therapeutic dose, suggesting that it could have a direct impact on gut microbiome composition.

To our knowledge, this is the first report of ABC activity against *G. vaginalis*. As AZT, ABC is a nucleoside analogue, for this reason we hypothesize that it could inhibit bacterial growth through DNA chain termination. Abacavir accumulation ratios in cervicovaginal secretions compared to blood plasma have been reported to be 0.08-0.58. Thus, considering the ABC peak plasma concentration at therapeutical doses, ABC concentrations in the female genital tract would be below the reported MICs against *G. vaginalis* (Else et al., 2011), frequently present in the vaginal microbiota (Klatt et al., 2017).

We identified EFV antibacterial activity against *P. bivia* and *G. vaginalis* with MIC values of 32 µg/mL. Ray et al. (Ray et al., 2021) identified EFV activity against *Prevotella* spp. agreeing with our findings, but also showed EFV activity against *Bacteroides* spp. and *E. faecalis* clinical strains which was not confirmed in our study using the reference strains. Additionally, Shilaih et al. (Shilaih et al., 2018) has previously identified EFV activity against *Bacillus subtilis* and Wallace et al. (Wallace et al., 2023) identified EFV activity against *B. cereus and E. coli*. Of note, EFV activity against *E. coli* was not observed in our study and previous research (Shilaih et al., 2018; Ray et al., 2021). However, to our knowledge, this is the first report of EFV antibacterial activity against *G. vaginalis.*

Reported gut lumen concentrations of EFV (Shilaih et al., 2018; Ray et al., 2021) are higher than the obtained MICs against the gut commensal *P. bivia*. However, EFV accumulation in cervicovaginal secretions has been reported to be minimal (0.25-0.34 ratio compared to blood plasma concentrations) (Else et al., 2011; Neely et al., 2015), thus according to our results, female genital concentrations of EFV would not reach antibacterial activity against *G. vaginalis*. Efavirenz has been a first-line ARV for more than 15 years and it is still used in low-and-middle income countries due to cost and availability although it is no longer recommended as a first-line treatment in high-income countries (Costa and Vale, 2022). Thus, EFV direct effect on gut microbiota composition could have an important impact in a great number of PLWH worldwide, as is the case of AZT, as mentioned before.

Finally, we report antibacterial activity of the integrase strand transfer inhibitors (INSTI) BIC and EVG. To our knowledge this is the first study to report BIC and EVG antibacterial activity. A previous study by Wallace et al. (Wallace et al., 2023) reported that DTG and Raltegravir (RAL) significantly reduced *E. coli* and *B. cereus* growth at concentrations ≥ 50 µg/mL. However, in our study we did not observe DTG and RAL antibacterial activity against tested *E. coli* strains. At present, INSTIs are part of the recommended first-line antiretroviral regimens in HIV treatment guidelines worldwide (World Health Organization, 2021; Palacios et al., 2023) being dolutegravir and BIC the most prescribed ARV among this family. Elvitegravir however, requires co-administration with cobicistat increasing the risk of drug-drug interactions, must be administered with food, has higher rates of adverse events and lower barrier to antiretroviral resistance. For these reasons, EVG is not part of most preferred ART regimens (Scarsi et al., 2020).

Previous studies on BIC rectal tissue and cervicovaginal fluid distribution show a 2.8% and 0.3-20% ratio to blood plasma, respectively (Imaz et al., 2021; Haaland et al., 2022), suggesting that, at the therapeutical dose, BIC would not reach antibacterial concentrations against gut and vaginal commensal microbiota. On the other hand, EVG accumulation in rectal and vaginal secretions have been observed to be 100- to 700-fold higher than plasma levels in a non-human primate model (Massud et al., 2014), and 10-fold higher in rectal tissues in a human model (Haaland et al., 2022). This suggests that EVG could reach gut and vaginal concentrations above the reported MICs against *G. vaginalis* (16 µg/mL), *P. bivia* (32 µg/mL), *Enterococcus* spp (4 µg/mL) and *S. aureus* (4 µg/mL).

More importantly, EVG could be repurposed for the treatment of infections caused by MDR Gram-positive bacteria as we report antibacterial activity against VRE and MRSA strains. The oral dosage of EVG to treat HIV infection is 150 mg combined with 150 mg of the pharmacokinetic enhancer cobicistat daily, and it is currently available co-formulated with emtricitabine and tenofovir disoproxil fumarate (TDF) (Genvoya^®^) or with emtricitabine and tenofovir alafenamide (TAF) (Stribild^®^). These formulations have been related to peak plasma concentration of 1,7-2,66 µg/mL (Gilead, 2021; Gilead, 2022; Podany et al., 2020) which would not reach plasma antibacterial activity. In previous studies where HIV-1 subjects were given unboosted EVG orally at doses ranging 200-800 mg, the maximum plasma concentration did not increase proportionally with the dose (DeJesus et al., 2006). Thus, further pharmacokinetic and safety studies on higher doses of boosted EVG should be performed to use EVG as an antibacterial agent.

Elvitegravir is a quinolone derivative INSTI, thus, it could share its antibacterial mechanism of action with quinolone antibiotics inhibiting DNA synthesis by targeting DNA gyrase and topoisomerase IV (Sato et al., 2006; Fàbrega et al., 2009). However, our results show that quinolone resistant isolates were not related to EVG resistance. On the other hand, Zhang et al. (Zhang et al., 2012) proposed that EVG derived compounds could inhibit bacterial growth targeting isoprenoid biosynthesis. These compounds showed antibacterial activity against Gram positive bacteria including *S. aureus* and *E. faecium* but little activity against *E. coli* showing a similar antibacterial profile as the identified in this study. Thus, another possible EVG mechanism of action could be targeting isoprenoid biosynthesis. Further studies are needed to elucidate EVG mechanism of action against Gram-positive bacteria and to investigate the pharmacological parameters that ensure the therapeutic efficiency *versus* the side effects associated with this ARV. In this context, the discovery of ARVs with antibacterial spectrum also raises important issues regarding the impact of ARV against the development of multidrug resistant microorganisms as well as regarding its ecotoxicological impact against the environment (Ncube et al., 2018; Wallace et al., 2023).

This study has limitations. As previously mentioned, there are differences between the antiretroviral antibacterial activity obtained in this study compared to the data presented by previous research (Ray et al., 2021; Wallace et al., 2023) probably due to the strains used; most of which being clinical isolates or different reference strains that might behave differently in the presence of the drug compared to the set of strains tested in our study. Additionally, MIC determination was tested using only one methodology and it could have been confirmed by other complementary techniques such as biomass evaluation by optical density, colony forming unit counting or DEAD/LIVE assays. Finally, additional commensal bacteria such as vaginal lactobacilli could have been included in the study to check the influence of the tested antiretroviral drugs on vaginal microbiota. Further studies are needed to externally validate our findings using more diverse clinical strains obtained from different origins.

In conclusion, we report *in vitro* antibacterial activity of extensively used antiretroviral drugs against gut and vaginal human commensal bacteria suggesting that ARV treatment could influence human microbiota composition. Finally, we report EVG antibacterial activity against MDR Gram-positive bacteria identifying its potential use as a new antibacterial agent.

## Data availability statement

The original contributions presented in the study are included in the article/**Supplementary Material**. Further inquiries can be directed to the corresponding author.

## Author contributions

ERG: Data curation, Formal Analysis, Methodology, Writing – original draft. NF: Data curation, Methodology, Writing – original draft. NM: Methodology, Writing – review & editing. CBD: Conceptualization, Funding acquisition, Supervision, Writing – review & editing. JM: Writing – review & editing. RP: Conceptualization, Funding acquisition, Project administration, Validation, Writing – review & editing. CCP: Conceptualization, Supervision, Validation, Writing – review & editing. JV: Conceptualization, Funding acquisition, Supervision, Validation, Writing – review & editing.

## References

[B1] Aira A. Fehér C. Rubio E. Soriano A. (2019). The intestinal microbiota as a reservoir and a therapeutic target to fight multi-drug-resistant bacteria: A narrative review of the literature. Infect. Dis. Ther. 8, 469–482. doi: 10.1007/s40121-019-00272-7 31654298 PMC6856238

[B2] Antonello R. M. di Bella S. Betts J. la Ragione R. Bressan R. Principe L. (2021). Zidovudine in synergistic combination with fosfomycin: an in *vitro* and in *vivo* evaluation against multidrug-resistant Enterobacterales. Int. J. Antimicrob. Agents 58, 106362. doi: 10.1016/j.ijantimicag.2021.106362 34010710

[B3] Gilead. (2022). Genvoya (Foster City, CA: Gilead Sciences, Inc). Available at: https://www.gilead.com/-/media/files/pdfs/medicines/hiv/genvoya/genvoya_pi.pdf.

[B4] Gilead. (2021). Stribild (Foster City, CA: Gilead Sciences). Available at: https://www.gilead.com/-/media/5b9b2d5ebf6a494a8730dfa627fda606.ashx.

[B5] Bandera A. Colella E. Rizzardini G. Gori A. Clerici M. (2017). Strategies to limit immune-activation in HIV patients. Expert Rev. Anti Infect. Ther. 15 (1), 43–54. doi: 10.1080/14787210.2017.1250624 27762148

[B6] Carpenter D. E. Anderson K. Citron D. M. Dzink-Fox J. L. Hackel M. Jenkins S. G. (2018). Clinical and laboratory standards institute (CLSI) methods for antimicrobial susceptibility testing of anaerobic bacteria. 9th Edition (Malvern, PA 19355: CLSI M11). Available at: https://clsi.org/standards/products/microbiology/documents/m11/.

[B7] Costa B. Vale N. (2022). Efavirenz: history, development and future. Biomolecules 13, 88. doi: 10.3390/biom13010088 36671473 PMC9855767

[B8] DeJesus E. Berger D. Markowitz M. Cohen C. Hawkins T. Ruane P. (2006). Antiviral activity, pharmacokinetics, and dose response of the HIV-1 integrase inhibitor GS-9137 (JTK-303) in treatment-naive and treatment-experienced patients. JAIDS J. Acquired Immune Deficiency Syndromes 43, 1–5. doi: 10.1097/01.qai.0000233308.82860.2f 16936557

[B9] Desarno A. E. Parcell B. J. Coote P. J. (2020). Repurposing the anti-viral drug zidovudine (AZT) in combination with meropenem as an effective treatment for infections with multi-drug resistant, carbapenemase-producing strains of Klebsiella pneumoniae. Pathog. Dis. 78, 1–10. doi: 10.1093/femspd/ftaa063 33053176

[B10] Doléans-Jordheim A. Bergeron E. Bereyziat F. Ben-Larbi S. Dumitrescu O. Mazoyer M. A. (2011). Zidovudine (AZT) has a bactericidal effect on enterobacteria and induces genetic modifications in resistant strains. Eur. J. Clin. Microbiol. Infect. Dis. 30, 1249–1256. doi: 10.1007/s10096-011-1220-3 21494911

[B11] Else L. J. Taylor S. Back D. J. Khoo S. H. (2011). Pharmacokinetics of antiretroviral drugs in anatomical sanctuary sites: The male and female genital tract. Antivir. Ther. 16, 1149–1167. doi: 10.3851/IMP1919 22155899

[B12] Elwell L. P. Ferone R. Freeman G. A. Fyfe J. A. Hill J. A. Ray P. H. (1987). Antibacterial activity and mechanism of action of 3’-azido-3’-deoxythymidine (BW A509U). Antimicrob. Agents Chemother. 31, 274–280. doi: 10.1128/AAC.31.2.274 3551832 PMC174705

[B13] Fàbrega A. Madurga S. Giralt E. Vila J. (2009). Mechanism of action of and resistance to quinolones. Microb. Biotechnol. 2 (1), 40–61. doi: 10.1111/j.1751-7915.2008.00063.x 21261881 PMC3815421

[B14] Falagas M. E. Voulgaris G. L. Tryfinopoulou K. Giakkoupi P. Kyriakidou M. Vatopoulos A. (2019). Synergistic activity of colistin with azidothymidine against colistin-resistant Klebsiella pneumoniae clinical isolates collected from inpatients in Greek hospitals. Int. J. Antimicrob. Agents 53, 855–858. doi: 10.1016/j.ijantimicag.2019.02.021 30836109

[B15] Guillén Y. Noguera-Julian M. Rivera J. Casadellà M. Zevin A. S. Rocafort M. (2019). Low nadir CD4+ T-cell counts predict gut dysbiosis in HIV-1 infection. Mucosal Immunol. 12, 232–246. doi: 10.1038/s41385-018-0083-7 30171206

[B16] Haaland R. E. Fountain J. Martin A. Dinh C. Holder A. Edwards T. E. (2022). Pharmacology of boosted and unboosted integrase strand transfer inhibitors for two-dose event-driven HIV prevention regimens among men. J. Antimicrob. Chemother. 78, 497–503. doi: 10.1093/jac/dkac419 PMC10161260 36512383

[B17] Henderson H. I. Napravnik S. Kosorok M. R. Gower E. W. Kinlaw A. C. Aiello A. E. (2022). Predicting risk of multidrug-Resistant enterobacterales infections among people with HIV. Open Forum Infect. Dis. 9 (10), ofac487. doi: 10.1093/ofid/ofac487 36225740 PMC9547514

[B18] Huemer M. Mairpady Shambat S. Brugger S. D. Zinkernagel A. S. (2020). Antibiotic resistance and persistence—Implications for human health and treatment perspectives. EMBO Rep. 21, e51034. doi: 10.15252/embr.202051034 33400359 PMC7726816

[B19] Imaz A. Tiraboschi J. M. Niubó J. Martinez-Picado J. Cottrell M. L. Domingo P. (2021). Dynamics of the decay of human immunodeficiency virus (HIV) RNA and distribution of bictegravir in the genital tract and rectum in antiretroviral-naive adults living with HIV–1 treated with bictegravir/emtricitabine/tenofovir alafenamide (Spanish HIV/AIDS research network, PreEC/RIS 58). Clin. Infect. Dis. 73, e1991–e1999. doi: 10.1093/cid/ciaa1416 32945851 PMC8492151

[B20] Klatt N. R. Cheu R. Birse K. Zevin A. S. Perner M. Noël-Romas L. (2017). Vaginal bacteria modify HIV tenofovir microbicide efficacy in African women. Science 356, 938–945. doi: 10.1126/science.aai9383 28572388

[B21] Lewin C. S. Allen R. A. Amyes S. G. B. (1990). Mechanisms of zidovudine resistance in bacteria. J. Med. Microbiol. 33, 235–238. doi: 10.1099/00222615-33-4-235 2124270

[B22] Lewin C. S. Watt B. Paton R. Amyes S. G. B. (2006). Isolation of zidovudine resistant Escherichia coli from AIDS patients. FEMS Microbiol. Lett. 70, 141–144. doi: 10.1111/j.1574-6968.1990.tb13967.x 2227349

[B23] Li S. X. Armstrong A. J. S. Neff C. P. Shaffer M. Lozupone C. A. Palmer B. E. (2016). Complexities of gut microbiome dysbiosis in the context of HIV infection and antiretroviral therapy. Clin. Pharmacol. Ther. 99, 600–611. doi: 10.1002/cpt.363 26940481 PMC4927263

[B24] Massud I. Martin A. Dinh C. Mitchell J. Jenkins L. Heneine W. (2014). Pharmacokinetic profile of raltegravir, elvitegravir and dolutegravir in plasma and mucosal secretions in rhesus macaques. J. Antimicrob. Chemother. 70, 1473–1481. doi: 10.1093/jac/dku556 PMC4398473 25630643

[B25] Ncube S. Madikizela L. M. Chimuka L. Nindi M. M. (2018). Environmental fate and ecotoxicological effects of antiretrovirals: A current global status and future perspectives. Water Res. 145, 231–247. doi: 10.1016/j.watres.2018.08.017 30142521

[B26] Neely M. Louie S. Xu J. Anthony P. Thuvamontolrat K. Mordwinkin N. (2015). Simultaneous plasma and genital pharmacokinetics and pharmacodynamics of atazanavir and efavirenz in HIV-infected women starting therapy. J. Clin. Pharmacol. 55, 798–808. doi: 10.1002/jcph.481 25683232 PMC4461473

[B27] Ng S. M. S. Sioson J. S. P. Yap J. M. Ng F. M. Ching H. S. V. Teo J. W. P. (2018). Repurposing Zidovudine in combination with Tigecycline for treating carbapenem-resistant Enterobacteriaceae infections. Eur. J. Clin. Microbiol. Infect. Dis. 37, 141–148. doi: 10.1007/s10096-017-3114-5 29019016

[B28] Olaru I. D. Ferrand R. A. Chisenga M. Yeung S. Macrae B. Chonzi P. (2021a). Prevalence of ESBL-producing Escherichia coli in adults with and without HIV presenting with urinary tract infections to primary care clinics in Zimbabwe. JAC Antimicrob. Resist. 3 (2), dlab082. doi: 10.1093/jacamr/dlab082 34223141 PMC8242135

[B29] Olaru I. D. Tacconelli E. Yeung S. Ferrand R. A. Stabler R. A. Hopkins H. (2021b). The association between antimicrobial resistance and HIV infection: a systematic review and meta-analysis. Clin. Microbiol. Infect. 27, 846–853. doi: 10.1016/j.cmi.2021.03.026 33813126

[B30] Palacios R. Ramón Arribas J. Polo R. González-García J. Bautista A. Antela A. (2023). Documento de consenso de GeSIDA/Plan Nacional sobre el Sida respecto al tratamiento antirretroviral en adultos infectados por el virus de la inmunodeficiencia humana (Actualización enero 2023). (Madrid, Spain: SEIMC).

[B31] Pinto-Cardoso S. Klatt N. R. Reyes-Terán G. (2018). Impact of antiretroviral drugs on the microbiome: Unknown answers to important questions. Curr. Opin. HIV AIDS 13, 53–60. doi: 10.1097/COH.0000000000000428 29028667 PMC5718259

[B32] Podany A. T. Scarsi K. K. Pham M. M. Fletcher C. V. (2020). Comparative clinical pharmacokinetics and pharmacodynamics of HIV-1 integrase strand transfer inhibitors: an updated review. Clin. Pharmacokinet. 59, 1085–1107. doi: 10.1007/s40262-020-00898-8 32462541 PMC7484053

[B33] Ray S. Narayanan A. Giske C. G. Neogi U. Sönnerborg A. Nowak P. (2021). Altered gut microbiome under antiretroviral therapy: impact of efavirenz and zidovudine. ACS Infect. Dis. 7, 1104–1115. doi: 10.1021/acsinfecdis.0c00536 33346662 PMC8154435

[B34] Sato M. Motomura T. Aramaki H. Matsuda T. Yamashita M. Ito Y. (2006). Novel HIV-1 integrase inhibitors derived from quinolone antibiotics. J. Med. Chem. 49, 1506–1508. doi: 10.1021/jm0600139 16509568

[B35] Scarsi K. K. Havens J. P. Podany A. T. Avedissian S. N. Fletcher C. V. (2020). HIV-1 integrase inhibitors: A comparative review of efficacy and safety. Drugs 80, 1649–1676. doi: 10.1007/s40265-020-01379-9 32860583 PMC7572875

[B36] Shilaih M. Angst D. C. Marzel A. Bonhoeffer S. Günthard H. F. Kouyos R. D. (2018). Antibacterial effects of antiretrovirals, potential implications for microbiome studies in HIV. Antivir Ther. 23, 91–94. doi: 10.3851/IMP3173 28497768

[B37] Unaids. (2022). Fact sheet. (CH-1211 Geneva 27 Switzerland: UNAIDS).

[B38] Wallace V. J. Sakowski E. G. Preheim S. P. Prasse C. (2023). Bacteria exposed to antiviral drugs develop antibiotic cross-resistance and unique resistance profiles. Commun. Biol. 6, 837. doi: 10.1038/s42003-023-05177-3 37573457 PMC10423222

[B39] Weinstein M. P. Patel J. B. Burnham C.-A. Campeau S. Conville P. S. Doern C. (2018). Clinical and laboratory standards institute (CLSI) methods for dilution antimicrobial susceptibility tests for bacteria that grow aerobically. 11th Edition (Malvern, PA 19355: CLSI; M07). Available at: https://clsi.org/standards/products/microbiology/documents/m07/.

[B40] World Health Organization. (2020) HIV/AIDS data and statistics. Available at: https://www.who.int/hiv/data/en/.

[B41] World Health Organization. (2021). Consolidated guidelines on HIV prevention, testing, treatment, service delivery and monitoring: recommendations for a public health approach. (Geneva: World Health Organization). 34370423

[B42] Zhang Y. Lin F. Y. Li K. Zhu W. Liu Y. L. Cao R. (2012). HIV-1 integrase inhibitor-inspired antibacterials targeting isoprenoid biosynthesis. ACS Med. Chem. Lett. 3, 402–406. doi: 10.1021/ml300038t 22662288 PMC3363326

