# Supplementary material for: In vitro antibacterial activity of antiretroviral drugs on key commensal bacteria from the human microbiota
Source: Front Cell Infect Microbiol. 2024 Jan 8;13:1306430. doi: 10.3389/fcimb.2023.1306430 (PMC10801051; doi:10.3389/fcimb.2023.1306430)
Supplement: Supplementary file 1 [file Table_1.docx]

**Supplementary Tables.**

**S1.** Antibiotic resistance profiles, sample origin and tested antiretroviral minimal inhibitory concentrations of the selected *Klebsiella pneumoniae* clinical strains. Antibiotic susceptibility testing was performed by the disk diffusion method following EUCAST guidelines.

| **Isolate number** | **AMP** | **AMC** | **CXM** | **FOX** | **CTX** | **TAZ** | **CEF** | **AZ** | **PTZ** | **IMI** | **MER** | **ERT** | **GEN** | **AMK** | **TOB** | **CIP** | **TEM** | **STX** | **Sample origin** | **Resistance mechanism** | **BIC MIC  (µg/mL)** |
| --- | --- | --- | --- | --- | --- | --- | --- | --- | --- | --- | --- | --- | --- | --- | --- | --- | --- | --- | --- | --- | --- |
| KP1 | R | R | R | R | R | R | R | R | R | R | R | R | R | R | S | R | R | R | Rectal swab | ESBL + OXA48 | >128 |
| KP2 | R | R | R | S | R | R | R | R | R | S | S | S | - | - | - | - | - | - | Rectal swab | ESBL | >128 |
| KP3 | R | R | R | S | R | R | R | R | R | S | S | S | R | S | R | R | S | R | Urine | ESBL | >128 |
| KP4 | R | S | S | S | S | S | S | S | S | S | S | S | S | S | S | S | S | S | Urine | - | 128 |
| KP5 | R | R | R | R | S | S | S | S | S | I | S | S | S | S | S | S | S | S | Urine | - | >128 |
| KP6 | R | S | S | S | S | S | S | S | S | S | S | S | S | S | S | S | S | S | Urine | - | >128 |
| KP7 | R | S | S | S | S | S | S | S | S | S | S | S | S | S | S | S | S | S | Wound swab | - | >128 |
| KP8 | R | S | S | S | S | S | S | S | S | S | S | S | S | S | S | S | S | S | Urine | - | >128 |
| KP9 | R | R | R | S | R | R | R | R | R | S | S | S | S | S | S | R | S | R | Urine | ESBL | >128 |
| KP10 | R | R | R | S | R | R | R | R | S | S | S | S | - | - | - | - | - | - | Rectal swab | ESBL | >128 |
| KP11 | R | R | R | S | R | R | R | R | R | S | S | S | R | S | R | R | S | R | Urine | ESBL | >128 |
| KP12 | R | R | R | R | R | R | R | R | R | R | R | R | R | R | R | R | R | R | Rectal swab | ESBL + OXA48 | >128 |
| KP13 | R | R | R | R | R | R | R | R | R | I | I | R | S | I | R | R | R | R | Rectal swab | ESBL + OXA48 | >128 |
| KP14 | R | R | R | R | R | R | R | R | R | S | S | R | - | - | - | - | - | - | Rectal swab | ESBL + OXA48 | >128 |
| KP15 | R | R | R | R | R | R | R | S | R | I | I | E | S | S | S | S | - | S | Rectal swab | NDM | >128 |
| KP16 | R | R | R | R | R | R | R | R | R | R | R | R | S | S | S | R | S | R | Rectal swab | KPC | >128 |
| KP17 | R | R | R | R | R | R | R | R | R | I | R | R | S | S | S | R | S | S | Rectal swab | KPC | >128 |

**S2.** Antibiotic resistance profiles, sample origin and tested antiretroviral minimal inhibitory concentrations of the selected *Escherichia coli* clinical strains. Antibiotic susceptibility testing was performed by the disk diffusion method following EUCAST guidelines.

| **Isolate number** | **AMP** | **AMC** | **CXM** | **FOX** | **CTX** | **TAZ** | **CEF** | **AZ** | **PTZ** | **IMI** | **MER** | **ERT** | **GEN** | **AMK** | **TOB** | **CIP** | **TEM** | **STX** | **Sample origin** | **Resistance mechanism** | **BIC MIC  (µg/mL)** |
| --- | --- | --- | --- | --- | --- | --- | --- | --- | --- | --- | --- | --- | --- | --- | --- | --- | --- | --- | --- | --- | --- |
| EC1 | R | R | S | S | S | S | S | S | S | S | S | S | S | S | S | S | S | S | Blood culture | - | >128 |
| EC2 | R | R | R | S | R | R | R | R | S | S | S | S | S | S | S | R | S | R | Blood culture | ESBL | 128 |
| EC3 | S | S | S | S | S | S | S | S | S | S | S | S | S | S | S | R | S | S | Blood culture | - | 128 |
| EC4 | S | S | S | S | S | S | S | S | S | S | S | S | S | S | S | S | S | S | Urine | - | >128 |
| EC5 | R | R | S | S | S | S | S | S | R | S | S | S | R | S | R | S | S | R | Urine | - | >128 |
| EC6 | R | R | R | S | R | R | R | R | S | S | S | S | S | S | S | R | I | R | Blood culture | ESBL | >128 |
| EC7 | S | S | S | S | S | S | S | S | S | S | S | S | S | S | S | S | S | S | Urine | - | >128 |
| EC8 | R | S | S | S | S | S | S | S | S | S | S | S | S | S | S | R | S | R | Urine | - | >128 |
| EC9 | S | S | S | S | S | S | S | S | S | S | S | S | S | S | S | S | S | S | Endometrial tissue | - | >128 |
| EC10 | S | S | S | S | S | S | S | S | S | S | S | S | S | S | S | S | S | S | Bile | - | >128 |
| EC11 | R | R | R | S | R | R | R | R | S | S | S | S | S | S | S | R | I | R | Blood culture | ESBL | 128 |
| EC12 | R | R | R | S | R | R | R | R | R | S | S | S | S | I | R | R | S | R | Abscess | ESBL | >128 |
| EC13 | R | R | R | S | R | R | R | S | S | S | S | S | S | S | S | R | S | S | Urine | ESBL | >128 |
| EC14 | R | R | R | S | R | R | R | R | S | S | S | S | S | S | S | S | S | S | Urine | ESBL | >128 |
| EC15 | R | R | R | S | R | R | R | R | S | S | S | S | R | S | S | R | S | R | Urine | ESBL | >128 |
| EC16 | R | R | R | R | R | R | R | R | R | R | R | R | S | S | S | R | R | R | Rectal swab | NDM | >128 |
| EC17 | R | R | R | S | S | S | S | R | R | S | S | R | S | S | S | S | S | S | Rectal swab | OXA-48 | 128 |
| EC18 | R | R | S | S | S | S | S | S | R | S | S | S | S | S | S | S | S | S | Rectal swab | OXA-48 | >128 |
| EC19 | R | R | R | R | R | R | R | R | R | R | R | R | R | S | R | R | R | R | Urine | ESBL+KPC | >128 |
| EC20 | R | R | R | R | R | R | R | S | R | R | S | R | S | I | R | R | R | R | Rectal swab | VIM | >128 |

**S3.** Antibiotic resistance profiles, sample origin and tested antiretroviral minimal inhibitory concentrations of the selected *Enterococcus faecalis* clinical strains. Antibiotic susceptibility testing was performed by the disk diffusion method following EUCAST guidelines.

| **Isolate number** | **AMP** | **VAN** | **TEI** | **LZ** | **LEV** | **NIT** | **Sample origin** | **BIC MIC  (µg/mL)** | **EVG MIC  (µg/mL)** |
| --- | --- | --- | --- | --- | --- | --- | --- | --- | --- |
| **EF1** | S | S | S | S | R | S | Urine | 64 | 8 |
| **EF2** | S | S | S | S | S | S | Urine | 64 | 16 |
| **EF3** | S | S | S | S | R | S | Urine | 64 | 16 |
| **EF4** | S | S | S | S | S | S | Peritoneal fluid | 64 | 8 |
| **EF5** | S | S | S | S | S | S | Wound swab | 64 | 4 |
| **EF6** | S | S | S | S | S | - | Abscess | 64 | 8 |
| **EF7** | S | S | S | S | S | - | Peritoneal fluid | 64 | 8 |
| **EF8** | S | S | S | S | S | - | Urine | 64 | 8 |
| **EF9** | S | S | S | S | S | S | Urine | 64 | 4 |
| **EF10** | S | S | S | S | R | S | Urine | 64 | 4 |
| **EF11** | S | S | S | S | R | S | Urine | 64 | 2 |
| **EF12** | S | S | S | S | R | S | Urine | 64 | 4 |
| **EF13** | S | S | S | S | S | S | Urine | 64 | 2 |
| **EF14** | S | S | S | S | S | S | Urine | 64 | 8 |
| **EF15** | S | S | S | S | R | S | Urine | 128 | 4 |
| **EF16** | S | S | S | S | S | S | Urine | 128 | 8 |

**S4.** Antibiotic resistance profiles, sample origin and tested antiretroviral minimal inhibitory concentrations of the selected *Enterococcus faecium* clinical strains. Antibiotic susceptibility testing was performed by the disk diffusion method following EUCAST guidelines.

| **Isolate number** | **AMP** | **VAN** | **TEI** | **LZ** | **LEV** | **NIT** | **Sample origin** | **Resistance mechanism** | **BIC MIC  (µg/mL)** | **EVG MIC  (µg/mL)** |
| --- | --- | --- | --- | --- | --- | --- | --- | --- | --- | --- |
| **EFA1** | R | S | S | S | R | - | Wound swab | - | 64 | 16 |
| **EFA2** | R | S | S | S | R | - | Abscess | - | 64 | 8 |
| **EFA3** | R | S | S | S | R | - | Ascitic fluid | - | 32 | 8 |
| **EFA4** | R | S | S | S | S | R | Urine | - | 64 | 16 |
| **EFA5** | R | S | S | S | R | S | Urine | - | 128 | 16 |
| **EFA6** | R | S | S | S | R | - | Intraabdominal collection | - | 64 | 2 |
| **EFA7** | R | S | S | S | R | S | Urine | - | 64 | 4 |
| **EFA8** | R | S | S | S | S | S | Urine | - | 64 | 8 |
| **EFA9** | - | S | - | - | - | - | Rectal swab | - | 32 | 8 |
| **EFA10** | - | S | - | - | - | - | Rectal swab | - | 64 | 8 |
| **EFA11** | R | S | S | S | - | - | Wound swab | - | 64 | 16 |
| **EFA12** | - | R | - | - | - | - | Rectal swab | VRE | 32 | 8 |
| **EFA13** | R | R | R | S | R | - | Wound swab | VRE | 64 | 4 |
| **EFA14** | - | R | - | - | - | - | Rectal swab | VRE | 32 | 4 |
| **EFA15** | - | R | - | - | - | - | Rectal swab | VRE | 32 | 4 |
| **EFA16** | - | R | - | - | - | - | Rectal swab | VRE | 32 | 4 |
| **EFA17** | R | R | R | S | - | - | Bile | VRE | 64 | 4 |
| **EFA18** | R | R | R | S | - | - | Ascitic fluid | VRE | 64 | 8 |
| **EFA19** | R | R | S | S | - | - | Rectal swab | VRE | 32 | 2 |
| **EFA20** |  | R | S | S | - | - | Rectal swab | VRE | 32 | 4 |
| **EFA21** | - | R | R | S | - | - | Rectal swab | VRE | 64 | 8 |
| **EFA22** | R | R | R | S | R | R | Urine | VRE | 64 | 4 |

**S5.** Antibiotic resistance profiles, sample origin and tested antiretroviral minimal inhibitory concentrations of the selected *Prevotella bivia* clinical strains. Antibiotic susceptibility testing was performed by the disk diffusion method following EUCAST guidelines.

| **Isolate number** | **Sample origin** | **AZT MIC  (µg/mL)** | **EFV MIC  (µg/mL)** | **EVG MIC  (µg/mL)** |
| --- | --- | --- | --- | --- |
| PB1 | External sample | 8 | 64 | 32 |
| PB2 | Perianal abscess | 4 | 64 | 32 |
| PB3 | Perianal abscess | 2 | 32 | 16 |
| PB4 | Wound aspirate | 2 | 32 | 16 |
| PB5 | Pilonidal abscess | 16 | 64 | 32 |
| PB6 | Placental swab | 4 | 128 | 32 |
| PB7 | Placental swab | 4 | 64 | 16 |
| PB8 | Endometrial aspirate | 4 | 32 | 32 |
| PB9 | Placental swab | 8 | 32 | 32 |
| PB10 | Blood culture | 8 | 64 | 32 |
| PB11 | Skin abscess | 8 | 64 | 16 |
| PB12 | Perianal abscess | 8 | 64 | 32 |

**S6.** Antibiotic resistance profiles, sample origin and tested antiretroviral minimal inhibitory concentrations of the selected *Gardnerella vaginalis* clinical strains. Antibiotic susceptibility testing was performed by the disk diffusion method following EUCAST guidelines.

| **Isolate number** | **Sample origin** | **ABC MIC  (µg/mL)** | **EFV MIC  (µg/mL)** | **BIC MIC  (µg/mL)** | **EVG MIC  (µg/mL)** |
| --- | --- | --- | --- | --- | --- |
| GV1 | Vaginal swab | 32 | 16 | 128 | 8 |
| GV2 | Vaginal swab | 64 | 16 | 128 | 16 |
| GV3 | Vaginal swab | 32 | 32 | 128 | 16 |
| GV4 | Vaginal swab | 32 | 16 | 128 | 8 |
| GV5 | Vaginal swab | 32 | 32 | 128 | 16 |
| GV6 | Vaginal swab | 64 | 32 | 128 | 8 |
| GV7 | Vaginal swab | >128 | 64 | >128 | 32 |
| GV8 | Vaginal swab | 32 | 32 | 128 | 16 |
| GV9 | Vaginal swab | 16 | 32 | 128 | 16 |
| GV10 | Vaginal swab | 16 | 32 | 128 | 8 |
| GV11 | Vaginal swab | 16 | 16 | 128 | 8 |
| GV12 | Vaginal swab | 16 | 32 | 128 | 16 |
| GV13 | Vaginal swab | 16 | 32 | 128 | 16 |
| GV14 | Vaginal swab | 16 | 32 | 128 | 8 |
| GV15 | Vaginal swab | 32 | 16 | 128 | 16 |
| GV16 | Vaginal swab | 32 | 16 | 128 | 16 |

**S7.** Antibiotic resistance profiles, sample origin and tested antiretroviral minimal inhibitory concentrations of the selected *Staphylococcus aureus* clinical strains. Antibiotic susceptibility testing was performed by the disk diffusion method following EUCAST guidelines.

| **Isolate number** | **LEV** | **CIP** | **MOX** | **Sample origin** | **Resistance mechanism** | **EVG MIC  (µg/mL)** |
| --- | --- | --- | --- | --- | --- | --- |
| SA1 | S | S | - | Blood | - | 4 |
| SA2 | S | S | - | Blood | - | 4 |
| SA3 | R | R | - | Abscess | MRSA | 4 |
| SA4 | S | R | - | Nasal swab | MRSA | 4 |
| SA5 | R | R | R | Blood | MRSA | 4 |
| SA6 | R | R | R | Facial wound | MRSA | 4 |
| SA7 | R | R | R | Muscle wound | - | 4 |
| SA8 | R | R | R | Groin | MRSA | 2 |
| SA9 | R | R | R | Wound | - | 4 |
| SA10 | - | - | - | - | - | 4 |
| SA11 | S | I | S | - | - | 4 |
| SA12 | I | R | - | Arm pus | MRSA | 4 |
| SA13 | R | R | - | Wound | MRSA | 4 |
| SA14 | I | R | - | Abdominal wound | MRSA | 4 |
| SA15 | R | R | R | Wound | MRSA | 4 |
| SA16 | R | R | R | Wound | - | 4 |
| SA17 | R | R | R | Wound | MRSA | 4 |
| SA18 | R | R | R | Sputum | - | 8 |
| SA19 | R | R | R | - | - | 4 |
| SA20 | I | S | S | Wound | - | 4 |
| SA21 | S | S | S | Bronchial | - | 4 |
| SA22 | R | R | R | Wound | - | 4 |

Abbreviations: ABC: Abacavir Sulfate, AMP: Amoxicllin, AMC: Amoxicillin/Clavulanic Acid, AMK: Amikacin, AZT: Zidovudine, BIC: Bictegravir, CIP: Ciprofloxacin, CTX: Ceftazidime, CXM: Cefuroxime, EFV: Efavirenz, EVG: EVGitegravir, ERT: Ertapenem, ESBL: extended-spectrum β-lactamase, FOX: Cefoxitine, GEN: Gentamicin, IMI: Imipenem, LEV: levofloxacin, LZ: Linezolid, MER: Meropenem, MOX: Moxifloxacin, MRSA: Methicillin-resistant *Staphylococcus* aureus, NIT: Nitrofurantoin, SXT: Cotrimoxazole, TAZ: Piperacillin/tazobactam, TEI: Teicoplanin, TEM: Temocillin, TOB: Tobramycin, VAN: Vancomycin, VRE: Vancomycin-resistant enterococci.
